# Characterization of a Species-Specific Insulinase-Like Protease in *Cryptosporidium parvum*

**DOI:** 10.3389/fmicb.2019.00354

**Published:** 2019-03-06

**Authors:** Shijing Zhang, Yuping Wang, Haizhen Wu, Na Li, Jianlin Jiang, Yaqiong Guo, Yaoyu Feng, Lihua Xiao

**Affiliations:** ^1^ State Key Laboratory of Bioreactor Engineering, School of Resources and Environmental Engineering, East China University of Science and Technology, Shanghai, China; ^2^ School of Biotechnology, East China University of Science and Technology, Shanghai, China; ^3^ College of Veterinary Medicine, South China Agricultural University, Guangzhou, China; ^4^ Emory Vaccine Center, Yerkes National Primate Research Center, Emory University, Atlanta, GA, United States

**Keywords:** *Cryptosporidium parvum*, insulinase, *cgd6_5520-5510*, invasion, expression

## Abstract

*Cryptosporidium parvum* is an intracellular protozoan that can cause severe diarrhea in humans and various mammals. Results of a comparative genomic analysis indicated that genes encoding two *C. parvum*-specific insulinase-like proteases (INS19 and INS20), *cgd6_5510* and *cgd6_5520*, are lost in many *Cryptosporidium* species. In this study, we provided evidence indicating that *cgd6_5510* and *cgd6_5520* are fragments of a full gene (*cgd6_5520-5510*) encoding one insulinase-like protease (INS20-19) that is similar in structure to classic insulinases. We expressed *cgd6_5510* in *Escherichia coli* for antiserum preparation and found the protein (INS19) that was partially degraded. A ~180 kDa protein of INS20-19 was specifically recognized by the polyclonal anti-INS19 antiserum in sporozoite lysate. We observed that INS20-19 is likely a protein with high expression in the apical region of sporozoites, and neutralization of the protein led to a partial reduction of parasite load in HCT-8 and MDBK cell cultures at 24 h. Taken together, our findings support the involvement of INS20-19 in the invasion or early developmental process of *C. parvum*.

## Introduction

*Cryptosporidium* spp. is significant causes of diarrhea in humans and various animals (Checkley et al., 2015). Immunocompetent individuals with cryptosporidiosis usually have acute illness, while immunocompromised hosts can have chronic and life-threatening diarrhea (Adamu et al., 2014). Between the two major human-pathogenic *Cryptosporidium* species, *Cryptosporidium hominis* has stricter host specificity, infecting mainly humans, nonhuman primates, and equine animals. In contrast, *Cryptosporidium parvum* has a broader host range, infecting humans as well as nonhuman primates, ruminants, equine animals, and some rodents, thus it is responsible for both anthroponotic and zoonotic transmission (Xiao, 2010). Currently, there are no effective vaccines, immunotherapies, or parasite-specific pharmaceuticals against *Cryptosporidium* infections (Bhalchandra et al., 2018).

Results of whole-genome characterization suggest that *C. parvum* and *C. hominis* differ by only ~3% in nucleotide sequences (Guo et al., 2015). Sequence differences between *C. parvum* and *C. hominis* genomes could provide clues on the genetic determinants of host specificity in *Cryptosporidium* spp. Most species-specific genes identified through comparative genomic analysis of *C. parvum* and *C. hominis* belong to two multigene families encoding the *Cryptosporidium*-specific MEDLE family of secreted proteins and insulinase-like proteases (INS). Among the latter, *cgd6_5510* and *cgd6_5520* (encoding proteases containing the M16 domain) are located in the sub-telomeric region of chromosome 6 of *C. parvum* and are absent in the *C. hominis* genome (Guo et al., 2015; Liu et al., 2016).

Insulinases are proteases containing the M16 domain, wildly distributed in prokaryotic and eukaryotic organisms, and characterized by an inverted Zn^2+^-binding motif (an active site with the consensus sequence His-Xaa-Xaa-Glu-His or “HXXEH”) within the first 200 amino acid residues of the N-terminus (Aleshin et al., 2009; Laliberté and Carruthers, 2011; Maruyama et al., 2011). Previous studies showed that insulinases have broad substrate specificity, cleaving and inactivating several small proteins and peptides (Qiu et al., 1998; Edbauer et al., 2002; Farris et al., 2005; Guo et al., 2010). In apicomplexans, falcilysin is a M16 insulinase involved in hemoglobin catabolism of *Plasmodium falciparum* (Murata and Goldberg, 2003). Toxolysins are M16 proteases of *Toxoplasma gondii* and act as maturases of microneme proteins or serve as regulators of such proteins during invasion. They are localized within the micronemes at the apical end of the parasites and thus are potentially involved in the egress, gliding motility or replication of parasites (Laliberté and Carruthers, 2011; Hajagos et al., 2012).

Little is known of *Cryptosporidium* insulinases. Comparative genomic analysis has detected 23 INS that contain the M16 domain in *C. parvum,* but only INS19 (encoded by *cgd6_5510*) and INS20 (encoded by *cgd6_5520*) are absent in *C. hominis* (Guo et al., 2015; Liu et al., 2016). As orthologs of *cgd6_5520* and *cgd6_5510* are present in *Cryptosporidium meleagridis* (with 86 and 88% nucleotide sequence identity, respectively), another *Cryptosporidium* species with a broad host range, INS encoded by these genes could potentially contribute to the broad host range of some *Cryptosporidium* spp.

In this study, we investigated the relationship between INS19 and INS20 and assessed their potential involvement in *C. parvum* infection of host cells.

## Materials and Methods

### *C. parvum* Oocysts and Preparation of Sporozoites and Sporozoite Lysate

Oocysts of the *C. parvum* IOWA isolate were purchased from Waterborne, Inc. (New Orleans, LA, United States). Prior to use, 3 × 10^7^ oocysts were treated with 0.5% sodium hypochlorite on ice for 10 min and washed three times with sterile PBS (pH 7.4) by centrifugation at 13,200 × *g* for 3 min. Hypochlorite-treated oocysts were excysted at 37°C for 30 min in the presence of 0.75% sodium taurocholate and 0.25% trypsin. The 8.5 × 10^7^ sporozoites generated were collected by centrifugation at 5,000 × *g* and 4°C for 10 min, washed three times with PBS at 13,200 × *g* and 4°C for 3 min, re-suspended in PBS, and used in *in vitro* culture. For the preparation of total proteins from sporozoites, 1 × 10^7^ hypochlorite-treated oocysts were excysted and protease inhibitors (Protease Inhibitor Cocktail Set III, EDTA-Free, Millipore, Billerica, MA, United States) added. The mixture of sporozoites and unexcysted oocysts was centrifuged at 13,200 × *g* for 3 min, and the pellet was washed with PBS and re-suspended in a lysis buffer (20 mM Tris buffer, pH 7.2, 135 mM sodium chloride, 10 mM manganese chloride, 1% Triton X-100, and protease inhibitor cocktail) as described (Bhalchandra et al., 2013). After incubation on ice for 2 h, the lysate was collected by centrifugation at 13,200 × *g* for 30 min. For the preparation of total proteins from oocysts, 1 × 10^7^ hypochlorite-treated oocysts were re-suspended in the lysis buffer and subjected to six freeze-thaw cycles. The lysate was collected by centrifugation at 13,200 × *g* for 30 min.

### Host Cells and Cell Culture

Human ileocecal adenocarcinoma HCT-8 cells and Madin-Darby bovine kidney (MDBK) cells were obtained from Chinese Academy of Science Shanghai Branch and grown to 90% confluence in 12-well plates (5 × 10^5^ cells/well) for qPCR analysis of gene expression or in 8-well chamber slides (1 × 10^5^ cells/well) for immunofluorescence detection of cultured parasites. The growth medium used was RPMI 1640 medium containing 10% fetal bovine serum, 1 mM sodium pyruvate, 50 U/ml penicillin G, 50 U/ml streptomycin, and 0.25 mg/ml amphotericin B (pH 7.4) (Gut and Nelson, 1999). In qPCR analysis, each well contained 5 × 10^5^ cells infected with 2 × 10^6^ sporozoites of *C. parvum*. In immunofluorescence detection, each well contained 1 × 10^5^ cells infected with 4 × 10^5^ sporozoites of *C. parvum*. The infected monolayers were cultured in maintenance medium (growth medium with 2% instead of 10% fetal bovine serum).

### Re-Annotation of the *cgd6_5520-5510* Gene

The original genome annotation of the *C. parvum* IOWA isolate identified *cgd6_5520* and *cgd6_5510* as two genes with some nucleotide sequence ambiguity. To obtain accurate nucleotide sequences of the genes, a fragment containing the full *cgd6_5520* and partial *cgd6_5510* (amplicon size = 1,978 bp) was amplified from DNA by PCR using primers designed based on upstream and downstream sequences: 5520-F91 (5′-AAAACCGCCAGCATACAAGA-3′) and 5510-R171 (5′-TGAGAGTGGAGCCCAGGTAT-3′). The intergenic region between *cgd6_5520* and *cgd6_5510* (amplicon size = 300 bp) was further amplified from cDNA by PCR using primers 5520-F1659 (5′-TACTAATTTAATACATCCTGA-3′) and 5510-R68 (5′-TGAATAATTTTAGTGTAAAG-3′). The template DNA was extracted from 1 × 10^7^ *C. parvum* oocysts using the Qiagen DNeasy Blood & Tissue Kit (Qiagen, Hilden, Germany), whereas total RNA was isolated from 2 × 10^6^ *C. parvum* sporozoites using the RNeasy Mini Kit (Qiagen) and used in cDNA synthesis using the RevertAid First Strand cDNA Synthesis Kit (Thermo Fisher Scientific, Waltham, MA, United States). The PCR analysis of DNA and cDNA was performed in duplicates with the Phusion High-Fidelity DNA Polymerase (Thermo Fisher Scientific) under the following conditions: denaturation at 95°C for 5 min; 35 cycles of amplifications at 95°C for 45 s, 50°C for 45 s, and 72°C for 60 s; and a final extension at 72°C for 7 min. The PCR products were sequenced, and the sequences generated were aligned with reference sequences (XM_001388322.1, XM_625315.1, XP_001388359.1, XP_625315.1, and nucleotides 1,325,091-1,328,460 of NC_006985) downloaded from GenBank by using the ClustalX version 2.0.11.^1^ The intergenic region between *cgd6_5520* and *cgd6_5510* was also analyzed using BLAST analysis of *C. parvum* RNA-Seq data ERX1790335, which were obtained from the Sequence Read Archive (SRA) of the National Center for Biotechnology Information (NCBI).^2^ RNA-Seq reads were mapped to the intergenic region using ClustalX. The prediction of glycosylation sites in the INS sequences was carried out using NetNGlyc^3^ and NetOGlyc.^4^ The full *cgd6_5520-5510* gene sequence was submitted to GenBank under accession number MK105815.

### Cloning and Expression of Recombinant INS19

The full-length *cgd6_5510* gene (XM_625315.1) was amplified by PCR from *C. parvum* genomic DNA using the Phusion High-Fidelity DNA Polymerase (Thermo Fisher Scientific). The primers used in the PCR were *Nco*I-F (5′-AAATCCATGGCCATGATGACTTTCATTTTCTTTTCTCT-3′) and *Xho*I-R (5′-AAATCTCGAGCTCATCTTTCATGAATTTGAGTTGG-3′, restriction enzyme sites underlined). The PCR amplification was performed under the following conditions: denaturation at 95°C for 5 min; 35 cycles of amplifications at 95°C for 45 s, 50°C for 45 s, and 72°C for 50 s; and a final extension at 72°C for 7 min. The PCR products were purified using a SanPrep Column PCR Product Purification Kit (Sangon Biotech, Shanghai, China), digested with *Nco*I and *Xho*I restriction enzymes (Thermo Fisher Scientific), and inserted into the expression vector pET28a (Novagen, Madison, WI, United States). *Escherichia coli* DH5α cells (Tiangen Biotech, Beijing, China) were transformed with the ligation products and grown in Luria-Bertani agar plates with 50 μg/ml kanamycin, with positive colonies being identified by PCR and sequencing. *Escherichia coli* Rosetta (DE3) cells (Tiangen Biotech) were transformed with the recombinant plasmid and cultured in Luria-Bertani medium supplemented with 50 μg/ml kanamycin and 34 μg/ml chloramphenicol. The induction of INS19 expression was performed by adding 0.5 mM isopropyl-β-D-thiogalactopyranoside (IPTG) at 16°C for 14 h.

### Purification of Recombinant INS19

Transformed *E. coli* Rosetta (DE3) cells were harvested by centrifugation and re-suspended in buffer A (10 mM Tris-HCl pH 8.0, 100 mM NaH_2_PO_4_, 0.1% Triton X-100, and 0.25 U/μl Benzonase nuclease). The cell suspension was supplemented with protease inhibitors and lysed with mild sonication at 4°C. Inclusion bodies containing the recombinant protein were collected by centrifugation at 13,200 × *g* and 4°C for 30 min and washed with buffer B (10 mM Tris-HCl pH 8.0, and 100 mM NaH_2_PO_4_) containing 1 or 2 M urea. The recombinant protein was solubilized in buffer B (10 mM Tris-HCl pH 8.0 and 100 mM NaH_2_PO_4_) containing 8 M urea, dialyzed against 10 mM Tris-HCl (pH 8.0), and concentrated by using Amicon^®^ Ultra-15 30 K Centrifugal Filter Devices (Millipore). The purified product was evaluated by using bicinchoninic acid assay (Enhanced BCA Protein Assay Kit, Beyotime, Shanghai, China) and sodium dodecyl sulfate polyacrylamide gel electrophoresis (SDS-PAGE, 10% acrylamide). Five milligrams of the purified protein was used in the preparation of polyclonal anti-INS19 antiserum in rabbits by GL Biochem Ltd. (Shanghai, China).

### Analysis of Recombinant INS19 Protein

Cell lysates or purification products were heated in reducing loading buffer at 95°C for 5 min, separated by 10% SDS-PAGE, and stained with Coomassie Brilliant Blue (Bio-Rad, Hercules, CA, United States). The expression of recombinant INS19 was assessed by the Western blot in which separated proteins were transferred from the SDS-PAGE gel to a polyvinylidene fluoride (PVDF) membrane at 400 mA and room temperature for 1 h using a semi-dry electro-blotting apparatus (Bio-Rad). They were incubated with anti-his-tag mAb (Cell Signaling Technology, Danvers, MA, United States) diluted 1:1,000 in blocking buffer (5% nonfat milk in TBST) at room temperature for 1 h, with nonspecific binding being blocked with pre-treatment of the membrane using the blocking buffer. After being washed three times with TBST, the PVDF membrane was incubated at room temperature for 1 h with peroxidase-conjugated goat anti-mouse IgG (H + L) antibody (Yeasen, Shanghai, China) diluted 1:5,000 in TBST, washed three times with TBST, and developed with High-sig ECL Western Blotting Substrate (Tanon, Shanghai, China) or the DAB kit (Tiangen Biotech). The purified INS19 protein and lysates from 8.5 × 10^7^ sporozoites and 1 × 10^7^ oocysts were further analyzed by the Western blot using polyclonal anti-INS19 antiserum (1:100) and pre-immune serum (1:100), with peroxidase-conjugated goat-anti-rabbit IgG (H + L) antibody (Yeasen) (1:5,000) being used as the secondary antibody in the Western blot analysis.

To verify the identity of degraded INS19 products, the bands for the truncated products (~53 and ~25 kDa) were excised from the SDS-PAGE gel and analyzed using matrix-assisted laser desorption/ionization time-of-flight mass spectrometry (MALDI-TOF/MS) (Applied Protein Technology, Shanghai, China).

### Assessment of *cgd6_5520-5510* Gene Expression in *C. parvum*

The relative transcription level of 3,302 genes, including *cgd6_5520* and *cgd6_5510*, in the *C. parvum* genome was assessed over a 72-h infection previously (Mauzy et al., 2012), which indicated some minor differences in gene expression levels between *cgd6_5520* and *cgd6_5510*. The data on *cgd6_5520* and *cgd6_5510* expression serve as positive controls for the present study of the relative expression level of the newly annotated *cgd6_5520-5510* gene in developmental stages of *C. parvum* using the same approach (Mauzy et al., 2012). Briefly, sporozoites were inoculated onto HCT-8 cell monolayers in 12-well plates at 2 × 10^6^ sporozoites/well and incubated at 37°C with 5% CO_2_ for 2 h. Free sporozoites were removed by washing the culture three times with PBS. The HCT-8 monolayers were further cultured in fresh maintenance medium. Total RNA was isolated from 2 × 10^6^ sporozoites and infected HCT-8 monolayers at 2, 6, 12, 24, 36, 48, and 72 h post-infection using the RNeasy Mini kit (Qiagen). Two micrograms of the total RNA was used in the cDNA synthesis using the RevertAid First Strand cDNA Synthesis Kit (Thermo Fisher Scientific). The expression of the *cgd6_5520-5510* gene was assessed using qPCR analysis of the cDNA with specific primers 5510-F490 5′-GGAAACATTCATCCTATT-3′ and 5510-R668 5′-CTAATCACTTTTGCGTAC-3′ (amplicon size = 179 bp), which were designed by using the Primer Premier 5 software.^5^ The expression of the *C. parvum* 18S rRNA gene (amplicon size = 255 bp) was used in data normalization, using primers Cp18s-995F (5′-TAGAGATTGGAGGTTGTTCCT-3′) and Cp18s-1206R (5′-CTCCACCAACTAAGAACGGCC-3′) (Cai et al., 2005; Mauzy et al., 2012). In qPCR, each 20-μl reaction mixture contained 1 μl of cDNA, 0.4 mM each primer, and 10 μl of 2 × SYBR Green Real-Time PCR Master Mix (Toyobo, Osaka, Japan). The amplification was conducted on a Light Cycler 480 (Roche, Basel, Switzerland), consisting of an initial denaturation at 95°C for 30 s and 40 cycles of 95°C for 5 s, 56°C for 10 s, and 72°C for 15 s. A melt curve analysis was performed between 57 and 95°C at the end of PCR amplification. The data were from duplicate qPCR analyses of cDNA from three independent experiments. The relative expression levels of *cgd6_5520-5510* gene in various developmental stages were calculated using the delta-delta method (Zhang et al., 2012).

### Assessment of INS20-19 Expression in Developmental Stages

For the assessment of INS20-19 expression in intracellular stages of *C. parvum*, HCT-8 cell layers were infected with 4 × 10^5^ sporozoites. After cultivation for 12, 24, and 48 h, they were fixed with 4% paraformaldehyde in PBS at room temperature for 30 min and treated with 0.5% Triton X-100 in PBS for 30 min. After blocking for nonspecific binding with 5% BSA in PBS at room temperature, the monolayers were probed with the polyclonal anti-INS19 antiserum. The HCT-8 monolayers on coverslips were stained with the Alexa Fluor 594-conjugated Anti-rabbit IgG (H + L) (Red) (Cell Signaling Technology), with the nuclei counter-stained with 4′, 6-diamidino-2-phenylindole (DAPI, Roche) in PBS. After washing with PBS, the coverslips were mounted with Anti-fade Mounting Medium (Boster, Wuhan, China) and examined with an Olympus BX53 microscope (Olympus, Tokyo, Japan). Oocysts and sporozoites fixed onto the SuperStick™ Slides (Waterborne) were stained with polyclonal anti-INS19 antiserum and examined similarly.

### *In vitro* Neutralization of Host Cell Invasion by *C. parvum*

To assess the involvement of INS20-19 in the broad host range of *C. parvum*, we employed both human- and bovine-origin cells (HCT-8 and MDBK cells, respectively) in neutralization by anti-INS19 antibodies. The effect of polyclonal anti-INS19 antiserum on *C. parvum* infection of HCT-8 and MDBK cells was examined by using an *in vitro* neutralization assay. HCT-8 or MDBK cells were seeded into 8-well chamber slides, grown to 90% confluence (1 × 10^5^ cells/well), washed with PBS, and infected with sporozoites at the level of 4 × 10^5^ sporozoites/well in antiserum or pre-immune serum diluted 1:1,000, 1:500, and 1:100 for 2 h. Before the infection, the sporozoites were pre-incubated at 37°C in 500 μl of antiserum or pre-immune serum diluted in maintenance medium for 30 min. After 2 h, the infected monolayers were washed off residual sporozoites and oocysts three times with PBS and further cultured in 500 μl of fresh maintenance medium for 24 h. Cultures infected with sporozoites in serum-free medium were used as controls to assess the inhibition of pre-immune serum itself, and uninfected cultures in serum-free medium were used to assess the background fluorescence signal. Afterward, the monolayers were washed with PBS and fixed with methanol. Cy3-labeled polyclonal anti-*C. parvum* antibody Sporo-Glo (Waterborne) was used to stain developmental stages of *C. parvum* as described (Upton et al., 1995; Deng et al., 2002). The slides were examined using immunofluorescence microscopy. Fifty random images were taken under 200 × magnification and analyzed by using ImageJ.^6^ The average number of parasites per 200 × field in each group was used to calculate the inhibition rate of *C. parvum* infection (Matsubayashi et al., 2013). The data obtained were expressed as mean ± standard deviation (SD) from three independent experiments. In this assay, treatment with the pre-immune serum served as another control.

### Statistical Analysis

Comparisons between treatment groups were done using the Student *t* test. Differences were considered significant at *p* ≤ 0.05.

## Results

### *cgd6_5510* and *cgd6_5520* Are Parts of a Full Gene *cgd6_5520-5510*

**Figure 1:**
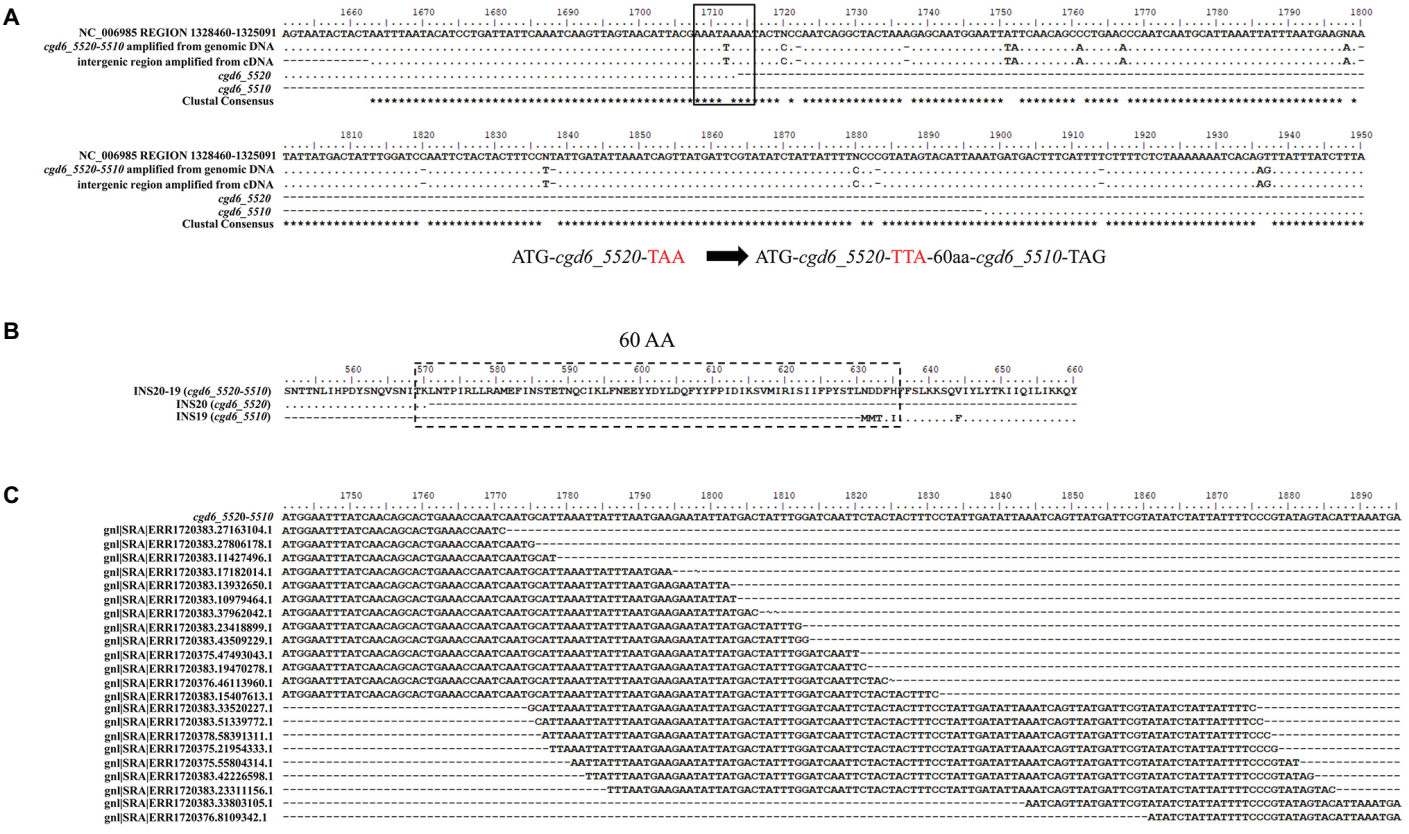
Evidence for the single gene nature of *cgd6_5510* and *cgd6_5520.* **(A)** Nucleotide sequence alignment of *cgd6_5520-5510* with the references *cgd6_5510* (XM_625315.1), *cgd6_5520* (XM_001388322.1), and nucleotides 1,325,091-1,328,460 of NC_006985. In the originally predicted intergenic region, there were seven nucleotide substitutions and seven nucleotide insertions, and the substitution of T by A (black box) at position 1,712 of the *cgd6_5520* in the reference genome had led to the introduction of a termination codon “TAA.” **(B)** Amino acid sequence alignment of INS20-19 (encoded by *cgd6_5520-5510*) with INS20 (encoded by *cgd6_5520*, XP_001388359.1) and INS19 (encoded by *cgd6_5510*, XP_625315.1) around the originally predicted intergenic region. In the new annotation, the translation continues until the termination codon of *cgd6_5510*, and there was an insertion of 60 amino acids (dashed box) between sequences encoded by *cgd6_5520* and *cgd6_5510*. **(C)** Mapping of *C. parvum* RNA-Seq reads to the intergenic region of the *cgd6_5520-5510* gene. The intergenic regions between *cgd6_5520* and *cgd6_5510* are nucleotides 1,714–1,891 of *cgd6_5520-5510.* RNA-Seq reads of ERX1790335 were downloaded from the NCBI SRA database and mapped to the intergenic region between *cgd6_5520* and *cgd6_5510*. The existence of overlapping reads indicates that the originally predicted intergenic region is indeed transcribed. All sequence alignments were manually adjusted for structurally equivalent by introducing gaps. The nucleotides or amino acids identical to the sequence in the reference (the first line of the alignment) are shown as dots in **A** and **B**.

The *cgd6_5520* (1,713 bp) was predicted to have a signal peptide and an active M16 peptidase domain at the N-terminus, while *cgd6_5510* (1,473 bp) did not have both. These two genes were supposedly located next to each other in the 3′ sub-telomeric region of chromosome 6, with 184 nucleotides between them. As they have amino acid sequence similarity to the first and second halves of normal insulinases, respectively, and there are nucleotide sequence ambiguities in the intergenic region between the two genes in the *C. parvum* reference genome, we attempted to determine whether these two genes are in fact one gene. After PCR amplification of the region covering the intergenic region between the two genes, comparison of DNA sequences obtained and the reference sequence from the *C. parvum* IOWA genome indicated that *cgd6_5520* and *cgd6_5510* were incorrectly annotated into two genes because of DNA sequencing errors (Figure 1A). In the originally predicted intergenic region, there were seven nucleotide substitutions, four unresolved nucleotides, and seven nucleotide insertions, all involving single-nucleotide repeats. In particular, the substitution of T by A at position 1,712 of *cgd6_5520* in the reference genome had led to the introduction of a termination codon “TAA.” In the new annotation, the translation continued until the termination codon of *cgd6_5510*. Altogether, there was an insertion of 60 amino acids between original *cgd6_5520* and *cgd6_5510* genes (Figure 1B).

The transcription of the intergenic region between *cgd6_5510* and *cgd6_5520* was confirmed by the analysis of cDNA and RNA-seq data from *C. parvum*. In PCR analysis of cDNA, a PCR product of the expected size (300 bp) was generated using primers (5520-F1659 and 5510-R68) flanking the intergenic region. DNA sequencing of the PCR product yielded a nucleotide sequence identical to the one generated from PCR analysis of the genomic DNA (Figure 1A). In the BLAST analysis of ERX1790335 from the NCBI SRA database, 68 RNA-Seq reads were mapped to the intergenic region between *cgd6_5520* and *cgd6_5510*, indicating the originally predicted intergenic region was indeed transcribed (Figure 1C).

Based on the results of these analyses, the newly annotated “*cgd6_5520-5510*” gene is 3,363 bp in length, has a few single-nucleotide polymorphisms (SNPs) and no frameshift insertion or deletion within the *cgd6_5510* region, and encodes a 1,120-amino acid insulinase-like protease “INS20-19.” Sixteen N-linked glycosylation sites and nine O-linked glycosylation sites were predicted in INS20-19. Six of these O-linked glycosylation sites and all but three of these N-linked glycosylation sites are located within the part encoded by *cgd6_5520*.

### Expression of the INS19 Fragment

We expressed the *cgd6_5510* fragment (INS19) rather than the full *cgd6_5520-5510* gene (INS20-19) considering that the region encoded by *cgd6_5510* is more specific and has no significant sequence homology to most *C. parvum* INS. In addition, we found that the full INS20-19 protein was poorly expressed in *E. coli*, and the recombinant produced was unstable, which made the affinity purification of the full protein difficult (unpublished data).

**Figure 2:**
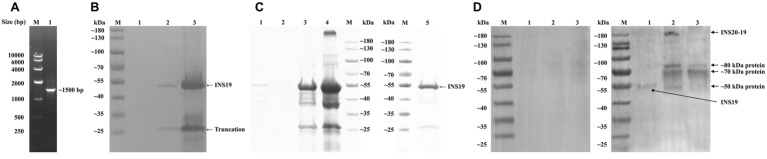
Production, purification, and characterization of the INS19 fragment expressed by *cgd6_5510*. **(A)** Electrophoresis analysis of a PCR product from the *cgd6_5510* region. Lane M: DNA marker; Lane 1: *cgd6_5510* PCR product. **(B)** The Western blot analysis of the expression of recombinant INS19 in *Escherichia coli* Rosetta (DE3). Lane M: protein marker; Lane 1: lysate of un-induced bacterial cells transformed with pET28s-*cgd6_5510*; Lane 2: supernatant from lysate of bacterial cells induced by IPTG; Lane 3: inclusion bodies from lysate of bacterial cells induced by IPTG. **(C)** Purification of recombinant INS19 in inclusion bodies as indicated by results of SDS-PAGE analysis. Lane M: protein marker; Lane 1: proteins from inclusion bodies dissolved in wash buffer containing 1 M urea; Lane 2: proteins from inclusion bodies dissolved in wash buffer containing 2 M urea; Lane 3: proteins from inclusion bodies dissolved in buffer containing 8 M urea; Lane 4: undissolved proteins from inclusion bodies after washing; Lane 5: purified recombinant INS19 after dialysis and ultrafiltration. **(D)** The Western blot analysis of purified INS19 (Lane 1), *C. parvum* sporozoite lysate (Lane 2), and *C. parvum* oocyst lysate (Lane 3) probed with the pre-immune serum (left panel) or polyclonal anti-INS19 antiserum (right panel). Lane M: protein marker.

The *cgd6_5510* fragment, which expresses an expected peptide of ~60 kDa, was amplified from genomic DNA byPCR (Figure 2A) and cloned into the expression vector pET28a. The recombinant protein (INS19) of *cgd6_5510* was mainly expressed in inclusion bodies and cleaved into two fragments of ~53 and ~25 kDa, as confirmed by the Western blot (Figure 2B) and MALDI-TOF/MS. We also constructed a pET41a-*cgd6_5510* expression vector that expressed a ~95 kDa recombinant INS19 with a GST-tag. The recombinant protein was also cleaved into fragments of ~53 and ~25 kDa (data not shown). The recombinant INS19 failed to bind to Ni-NTA resins despite the presence of 6× his-tag (data not shown). The SDS-PAGE analysis showed that the ~53 kDa protein constituted of over 80% proteins in inclusion bodies, and most of the recombinant proteins could be dissolved in 8 M urea. After dilution, dialysis, and ultrafiltration, the purity of the refolded protein reached ~90% (Figure 2C). The recombinant INS19 expressed by the *cgd6_5510* fragment was recognized by the polyclonal anti-INS19 antiserum from rabbits immunized with the purified recombinant protein, but not by the pre-immune serum. The polyclonal anti-INS19 antiserum specifically recognized the ~180 kDa full-length INS20-19 protein in *C. parvum* sporozoites and three fragments of ~50, ~70, and ~80 kDa in *C. parvum* sporozoites and oocysts (Figure 2D).

### Expression of the *cgd6_5520-5510* Gene in *C. parvum* Culture

**Figure 3:**
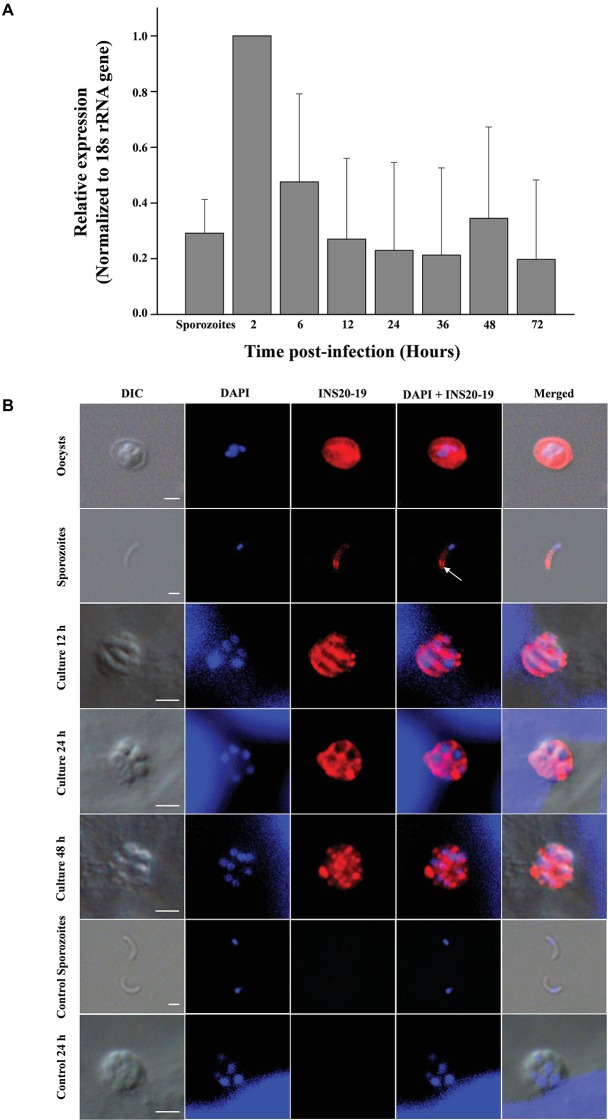
Expression of the *cgd6_5520-5510* gene and its product INS20-19 in different development stages of *C. parvum*. **(A)** Relative expression level of *cgd6_5520-5510* gene over a 72-h time course in *C. parvum*-infected HCT-8 cells as indicated by qPCR analysis. Bars represent standard deviation of the means from three individual experiments with similar results. In each experiment, we calculated the fold difference relative to the 2^-ΔΔCt^ value of 2 h post-infection. Thus, the relative expression level at 2 h post-infection is 1 with no standard deviation. The expression of the *cgd6_5520-5510* gene was the highest at 2 h post-infection and declined thereafter. **(B)** The expression of INS20-19 in oocysts, sporozoites, and intracellular stages of *C. parvum* in HCT-8 cells as indicated by immunofluorescence microscopy. The reactivity of the polyclonal anti-INS19 antiserum with oocysts, free sporozoites, and merozoites in infected HCT-8 cells is shown (red). Nuclei were counter-stained with DAPI (blue). Apical and surface region of sporozoites are indicated with arrows. No signal was detected in parasites incubated with the pre-immune serum (not shown). Scale bars = 2 μm.

The relative expression level of the *cgd6_5520-5510* gene over a 72-h time course in *C. parvum*-infected HCT-8 cells was assessed by qPCR. The expression of the *cgd6_5520-5510* gene was the highest at 2 h post-infection and declined thereafter (Figure 3A). This was similar to the published data on the expression of the original *cgd6_5520* and *cgd6_5510* genes, especially data from the former (Mauzy et al., 2012).

### Expression of INS20-19 Protein in Developmental Stages of *C. parvum*

An indirect immunofluorescence assay was used to examine the expression of INS20-19 in oocysts, sporozoites, and intracellular stages of *C. parvum*. In oocysts, polyclonal anti-INS19 antiserum apparently reacted with all contents, including the sporozoites. In sporozoites, the antiserum recognized predominantly the surface of the apical region (Figure 3B). In *C. parvum-*infected HCT-8 cultures, we observed both type I meronts containing eight merozoites and type II meronts containing four merozoites (Bouzid et al., 2013) at 12, 24, and 48 h post-infection using the combination of differential interference contrast (DIC) microscopy and nucleus staining with DAPI (blue). The antiserum reacted with the entire merozoites at 12 and 24 h post-infection, but not with the parasitophorous vacuole membrane. At 48 h post-infection, the fluorescence signal of INS20-19 on merozoites was more dispersed. In contrast, the pre-immune serum did not react with the oocysts, sporozoites, and developing parasites in culture (Figure 3B).

### Polyclonal Anti-INS19 Antiserum Partially Neutralized *C. parvum* Infection of HCT-8 and MDBK Cells

**Figure 4:**
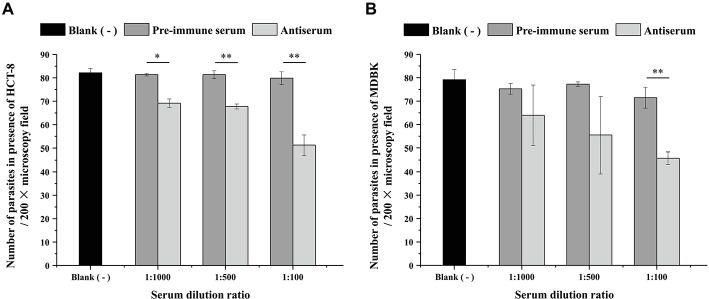
Effect of the polyclonal anti-INS19 antiserum on *C. parvum* infection of HCT-8 cells **(A)** and MDBK cells **(B)**. Cultures infected with sporozoites in polyclonal anti-INS19 antiserum or pre-immune serum diluted 1:1,000, 1:500, and 1:100 were treatment groups, whereas cultures infected with sporozoites in serum-free medium were used as controls. In each group, 50 random 200 × fields in immunofluorescence microscopy were analyzed for parasite load. The average number of parasites per field in each group was used to calculate the inhibition rate of *C. parvum* invasion. Each bar represents the mean ± S.D. of three independent experiments. *p < 0.05; **p < 0.01.

An *in vitro* neutralization assay was used to evaluate the effect of the polyclonal anti-INS19 antiserum on *C. parvum* infection. The parasite load was measured at 24 h of the culture by direct immunofluorescence microscopy. In the HCT-8 invasion assay, the mean numbers of parasites were 69.2 ± 1.82, 67.8 ± 1.08, and 51.3 ± 4.31 per 200 × field in cultures treated with the antiserum diluted 1:1,000, 1:500, and 1:100, respectively, compared with 81.4 ± 0.58, 81.4 ± 1.67, and 79.9 ± 2.72 per 200 × field in cultures treated with the pre-immune serum diluted 1:1,000, 1:500, and 1:100, respectively. In the culture infected with sporozoites in serum-free medium, the number of parasites per 200 × field was 82.1 ± 1.92. Thus, the polyclonal anti-INS19 antiserum reduced *C. parvum* infection of HCT-8 cells by 15.0% (*t*_(2)_ = 8.874, *p* = 0.012), 16.7% (*t*_(2)_ = 10.854, *p* = 0.008), and 35.8% (*t*_(2)_ = 11.817, *p* = 0.007) at 1:1,000, 1:500, and 1:100 dilutions, respectively, compared with data from cultures treated with the pre-immune serum (Figure 4A). In the MDBK cell culture, the mean numbers of parasites were 64.0 ± 12.90, 55.5 ± 16.45, and 45.8 ± 2.66 per 200 × field in cultures treated with the antiserum diluted 1:1,000, 1:500, and 1:100, respectively, compared with 75.2 ± 2.31, 77.2 ± 1.01, and 71.5 ± 4.45 per 200 × field in cultures treated with the pre-immune serum diluted 1:1,000, 1:500, and 1:100, respectively. In the culture infected with sporozoites in serum-free medium, the number of parasites per 200 × field was 79.2 ± 4.35. Thus, the polyclonal anti-INS19 antiserum reduced *C. parvum* infection of MDBK cells by 14.9% (*t*_(2)_ = 1.835, *p* = 0.208), 28.1% (*t*_(2)_ = 2.227, *p* = 0.156), and 35.9% (*t*_(2)_ = 20.784, *p* = 0.002) at 1:1,000, 1:500, and 1:100 dilutions, respectively, compared with data from cultures treated with the pre-immune serum (Figure 4B).

## Discussion

Data obtained in the study suggest that *cgd6_5510* and *cgd6_5520* annotated in the originally published *C. parvum* genome are in fact two fragments of a larger gene whose amino sequence is similar in structure to other INS in *C. parvum*. This mis-annotation could have resulted from sequence ambiguity in the putative intergenic region between the two genes in the published *C. parvum* IOWA genome. This had led to the erroneous introduction of a termination codon at the end of *cgd6_5520*, four unresolved nucleotides and six single-nucleotide insertions in the intergenic region, and one single-nucleotide insertion in the 5′ end of *cgd6_5510*. As a complete *C. parvum*-specific gene, *cgd6_5520-5510* encodes INS20-19 that has peptidase family M16 domains in the *cgd6_5520* part and a more specific *cgd6_5510* region.

The function of INS20-19 in *C. parvum* is not clear. However, it is probably not a classic insulinase. A complete insulinase such as the human insulinase usually has four conserved domains. Among them, the N-terminal domain contains the inverted Zn^2+^-binding motif “HXXEH,” a key feature of M16 proteases, while the C-terminal domain is also required for dimerization and substrate recognition (Johnson et al., 2006; Li et al., 2006). However, INS20-19 in *C. parvum* has the “HLLKQ” sequence instead of the core motif “HXXEH” and has only two of the four domains of functional insulinases. Results of a previous study indicated that mutants with other motif sequences such as “HFCQH” have no proteolytic activities (Chesneau and Rosner, 2000). Compared with other INS genes that mostly have the highest expression at later time points of the *C. parvum* infection (Mauzy et al., 2012), the *cgd6_5520-5510* gene is highly expressed during early development of parasites in cell cultures, supporting the potential involvement of INS20-19 in the invasion or early developmental process of *C. parvum*.

In this study, we expressed the INS19 encoded by the *cgd6_5510* fragment of the gene and found the recombinant INS19 that was mainly expressed in inclusion bodies and degraded into two fragments of ~53 and ~25 kDa in *E. coli* lysate. In the Western blot analysis, the ~180 kDa full-length INS20-19 protein was detected in crude extract from *C. parvum* sporozoites but not in the extract from oocysts, suggesting there was biosynthesis of the full-length protein during excystation. As proteins of ~50, ~70, and ~80 kDa were recognized by the polyclonal anti-INS19 antiserum in both sporozoite and oocyst extracts, INS20-19 appears to be proteolytically processed in *C. parvum*. In previous studies, toxolysins of *T. gondii* were shown to be proteolytically processed at the highly conserved SФXE (in which Φ is hydrophobic and X is any amino acid) site (Laliberté and Carruthers, 2011; Hajagos et al., 2012). However, the ~50 kDa species of INS20-19 in *C. parvum* sporozoites might not have resulted from a similar cleavage because peptides of ~40, ~60, ~70, ~80, and ~100 kDa rather than ~50, ~70, and ~80 kDa would be formed through cleavage at the SФXE site of INS20-19, as INS19 has few glycosylation sites. Therefore, INS20-19 could be proteolytically processed differently from M16 proteases of *T. gondii*. It remains to be determined whether INS20-19 acts as a maturase of other proteinases or serves as a precursor/regulator of such proteins during invasion.

The excystation of the oocysts requires the involvement of zinc-binding aminopeptidases (Okhuysen et al., 1996; Snelling et al., 2007; Singh et al., 2015). The sporozoites in the thick-walled oocysts emerge through a suture in the oocyst wall (Hijjawi, 2010; Bouzid et al., 2013), and the infectious sporozoites glide over the host cells, releasing material from the apical complex and depositing trails of proteins during invasion (Okhuysen and Chappell, 2002; Wanyiri and Ward, 2006). Many of these *Cryptosporidium* surface and apical complex proteins, such as GP40, GP15, P23, and Cp47, are involved in the attachment and invasion of *C. parvum* (Wanyiri and Ward, 2006). INS20-19 is apparently excreted in large quantity into the oocyst in addition to its presence on the surface of the apical region of sporozoites, supporting its potential involvement in oocyst excystation and sporozoite invasion. As INS20-19 is also expressed on the entire merozoites in large quantity, it could also be involved in the infection of epithelial cells by merozoites.

In this study, polyclonal anti-INS19 antiserum partially neutralized *C. parvum* infection of HCT-8 and MDBK cells *in vitro*, supporting the potential involvement of INS20-19 in invasion. Previously, the entry of *C. parvum* into HCT-8 cells was shown to be more efficient than *C. hominis* (Hashim et al., 2004, 2006), and apicomplexans have been known to use multiple strategies for invasion (Sibley, 2004). As *C. hominis* has no *cgd6_5520-5510* gene but can infect HCT-8 cells, the partial neutralization effect of the polyclonal anti-INS19 antiserum is expected. The similar neutralization patterns of HCT-8 and MDBK cells by antibodies suggest that if INS20-19 is directly involved in the invasion process of *C. parvum* through processing host proteins, its targets are present in both bovine and human epithelial cells. In an earlier study, toxolysins of *T. gondii* were shown to be required for parasite fitness (Laliberté and Carruthers, 2011). It would be interesting to determine whether INS20-19 is associated with the fitness of *C. parvum*. Further studies are required to investigate whether the neutralization of INS20-19 could lead to a reduction of parasite load *in vivo*.

In summary, results of the preliminary study suggest that INS20-19 is probably involved in the invasion or early developmental process of *C. parvum*. The potential involvement of INS20-19 in *C. parvum* infection of host cells raises the possibility that it could be a potential target for immunological or therapeutic interventions. Further studies using more advanced technologies such as confocal and immuno-electron microscopy and genetic manipulation are needed to determine fully the subcellular location and mechanism of INS20-19 in the initial host-parasite interactions by *C. parvum*.

## Author Contributions

YF and LX conceived and designed the experiments. SZ and YW performed the experiments. HW, NL, and JJ provided technical assistance. SZ, YW, YF, and LX analyzed the data. SZ, YF, and LX wrote the manuscript. All authors read and approved the final manuscript.

### Conflict of Interest Statement

The authors declare that the research was conducted in the absence of any commercial or financial relationships that could be construed as a potential conflict of interest.

## Footnotes

^1^http://www.clustal.org

^2^http://trace.ncbi.nlm.nih.gov/Traces/sra/sra.cgi

^3^http://www.cbs.dtu.dk/services/NetNGlyc/

^4^http://www.cbs.dtu.dk/services/NetOGlyc/

^5^http://www.premierbiosoft.com/crm/jsp/com/pbi/crm/clientside/ProductList.jsp

^6^https://imagej.nih.gov/ij/download.html
